# Ewing’s sarcoma of the male external genitalia: a case report and review of the literature

**DOI:** 10.1186/s12894-022-01072-x

**Published:** 2022-08-08

**Authors:** Sruti Rachapudi, Aditya Srinivasan, Brendan Gorman, Alyeesha B. Wilhelm, Eduardo Eyzaguirre, Eduardo Orihuela

**Affiliations:** 1 School of Medicine, University of Texas Medical Branch, Galveston, TX USA; 2 Division of Urology, Department of Surgery, University of Texas Medical Branch, 301 University Blvd, Galveston, TX 77555 USA; 3 Department of Pathology, University of Texas Medical Branch, Galveston, TX USA

**Keywords:** Paratesticular neoplasms, Ewing sarcoma, Extraosseous Ewing sarcoma, Genitourinary system, Urology

## Abstract

**Background:**

Ewing’s sarcoma (ES) within the genitourinary tract are relatively unheard of and those within the external male genitalia are even rarer. To our knowledge, this is the first known case of primary ES within the paratesticular region in an adult.

**Case presentation:**

We present a case of a 24-year-old man with a right sided testicular mass on examination that was initially characterized as an adenomatoid tumor on ultrasound. After the patient was lost to follow up over the course of 9 months, the testicular mass grew significantly and was excised with pathology revealing primary paratesticular Ewing’s sarcoma. This rare case emphasizes the importance of elucidating between the broad differentials of paratesticular masses, including the rare presentation of primary ES and adds a review of the literature of ES in the external male genitalia.

**Conclusions:**

Rare differentials such as this case should be considered in patients with paratesticular masses. Further diagnostic and management algorithms for extraosseous Ewing Sarcoma, particularly in the adult population, are warranted.

## Background

Primary paratesticular malignancies are rare, and sarcoma paratesticular malignancies are even more so. The paratesticular region contains the spermatic cord, testicular tunica layers, and the epididymis. Sarcomas of the genitourinary origin are exceedingly rare, and constitute approximately 2.1% of soft tissue sarcomas and 1–2% of malignant genitourinary tumors [1, 2]. Within the genitourinary tract, the most common histological subtypes are leiomyosarcoma, liposarcoma and rhabdomyosarcoma, which affected the paratesticular region, kidney, prostate, penis and bladder, in descending order [3]. Other rarer sarcomas are possible, including extraosseous Ewing Sarcoma (ES) which represents 6–24% of all ES cases [4]. There is currently one known report in the literature of ES in the scrotum of a pediatric patient [5]. We present, to the best of our knowledge, the first reported adult case of paratesticular ES—initially appearing as an adenomatoid tumor on ultrasound—and the second reported case overall and additionally reviewed the literature regarding reported cases of ES in the male external genitalia.

## Case presentation

**Fig. 1:**
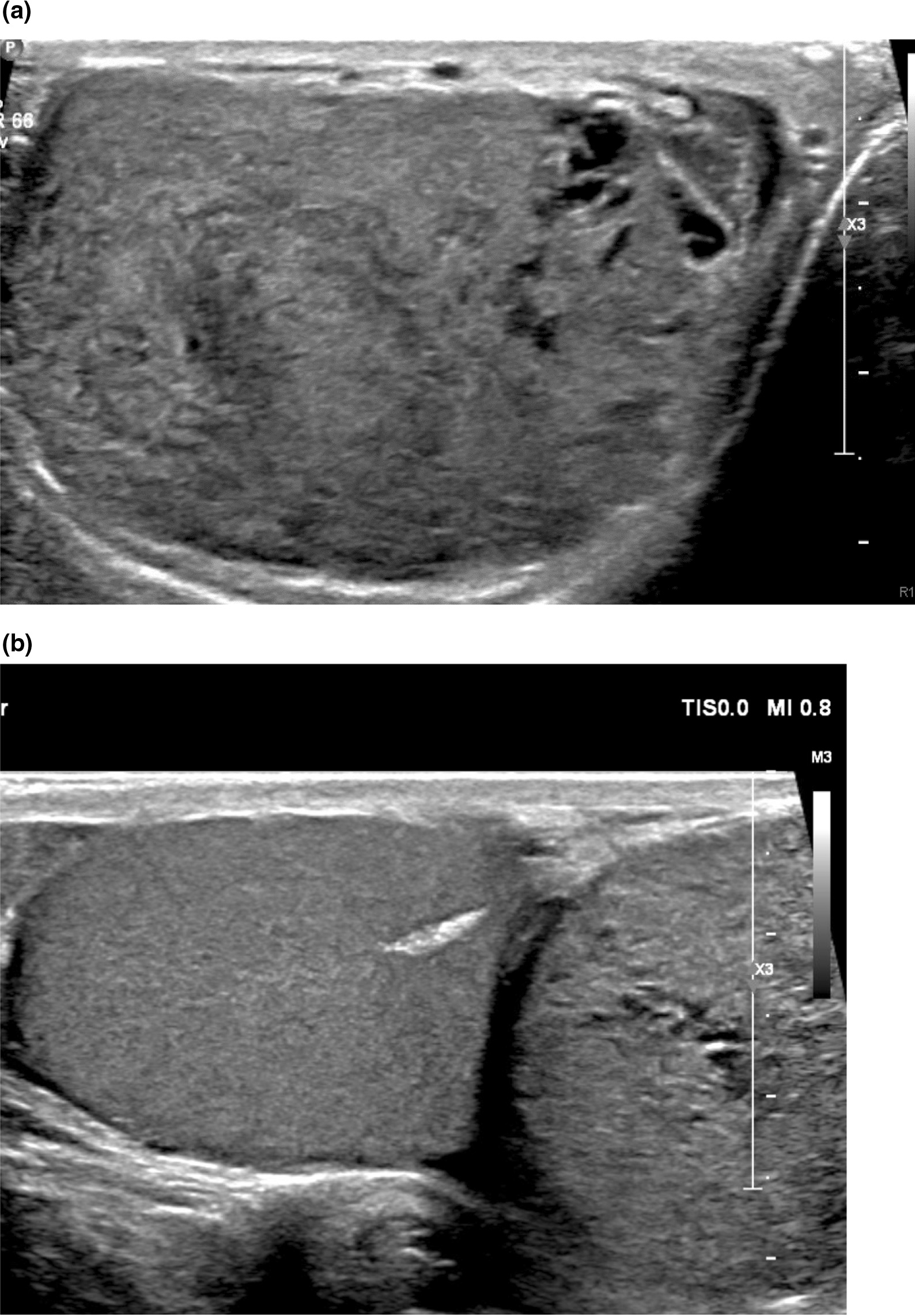
**a** (top image) Extratesticular mass on ultrasound in the right hemiscrotum, **b** (bottom image) side-by-side views of both testicle (left) and part of extratesticular mass (right)

A 24-year-old Hispanic male with no prior medical or surgical history presented with a right-sided testicular mass and pain for 3 months. Upon presentation, the patient endorsed continued right-sided testicular pain and increased size of the testicular mass. Physical exam revealed an uncircumcised penis, normal phallus, bilateral descended testes with a large right testicular soft, tender, and mobile mass. Testicular ultrasound at this time showed an extratesticular 4 cm (cm) mass that, per radiologic interpretation, resembled an adenomatoid tumor. He was scheduled for surgery but lost to follow-up. Nine months later, he presented with continued pain and increased size of the right hemiscrotum with separately palpated and larger extratesticular mass on physical exam and ultrasound (Fig. 1). Tumor markers were drawn: AFP and beta-hCG were within normal limits, and LDH was mildly elevated (634 units/liter). The patient, however, was unfortunately lost to follow-up again and his surgery was finally rescheduled 1 year and 4 months after his initial presentation. On final clinic follow-up before surgery, his right hemiscrotum had increased significantly in size, and there was growing suspicion for a malignant etiology of the tumor.

Intraoperatively, an inguinoscrotal incision was used to deliver the testicle within the spermatic fascia and a firm 8 cm paratesticular mass was palpated and concerning for malignancy. The testicle was indiscernible from the mass. A radical orchiectomy was performed.

**Fig. 2:**
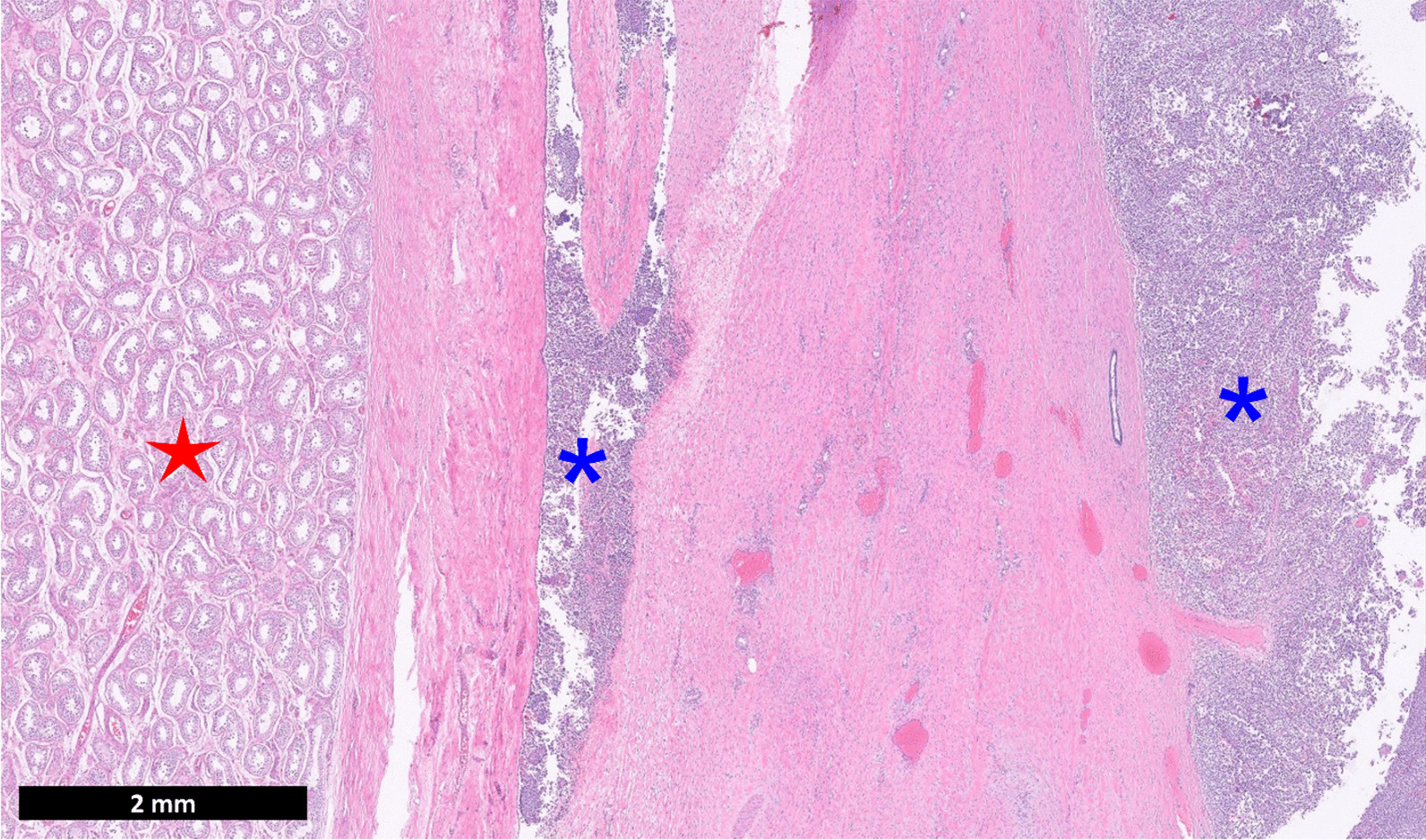
Histopathology revealing Ewing sarcoma (blue asterisk) near the uninvolved testicular parenchyma (red star) (H&E stain)

Final histopathology revealed a 7.3 cm × 6.5 cm × 6.0 cm paratesticular mass arising from the parietal tunica vaginalis of the testicle and extending into the visceral tunica vaginalis and epididymis (Fig. 2). The tumor did not extend outside of the spermatic fascia and margins were negative for malignancy. LDH subsequently normalized to 403 units/liter two weeks after surgery. A subsequent computed tomography (CT) scan of the thorax and nuclear medicine bone scan showed no evidence of metastatic disease. Immunohistochemical stains showed the tumor cells to be diffusely positive for CD99 (membranous) and negative for keratin, WT1 and neuron specific enolase (NSE). FISH for EWSR1 gene rearrangement was positive, confirming the diagnosis. The patient is currently being treated with 7–8 cycles of adjuvant chemotherapy (vincristine, doxorubicin, and cyclophosphamide alternating with ifosfamide and etoposide). The patient remains free of metastases four months after initial diagnosis.

## Discussion and conclusions

Due to the clinically silent nature of paratesticular tumors before a noticeable mass effect, it is challenging to diagnose them. Differentials for such masses include benign lesions such as lipomas, hemangiomas, lymphangiomas, leiomyomas [6] and adenomatoid tumors which account for 30% of all paratesticular masses [7]. Malignant differentials include liposarcoma, leiomyosarcoma, and rhabdomyosarcoma. Other rare paratesticular differentials include ovarian-type tumors arising from Mullerian metaplasia of the tunica vaginalis, desmoplastic small cell tumors, and melanotic neuroectodermal tumors [8]. Although ES has a propensity for extraosseous presentations, the reason for this affinity is relatively unknown. Further research is warranted to evaluate the pathophysiology of extraskeletal ES and to determine if genetic testing can play a role in the workup of paratesticular malignancies.

Scrotal sarcomas are rare and are commonly liposarcomas, rhabdomyosarcomas, or leiomyosarcomas [6]. It has been suggested that for these tumors, a CT or magnetic resonance imaging (MRI) scan is preferred to diagnose liposarcoma due to the presence of abnormal fat appearing with unreliable echogenicity on ultrasound [9]. It is also posited that MRI can provide a better characterization of tissue that may correlate with the histologic type of testicular tumor [10, 11]. Diagnostic precision may be greater with MRI, not only in testicular masses but also in spermatic cord masses [12]. However, for other tumors such as rhabdomyosarcoma, leiomyosarcoma, and ES, their imaging appearance is non-specific, often showing increased vascularity on ultrasound (US) or variable heterogenous enhancement on MRI [9]. Interestingly, the original US, in combination with relatively normal tumor markers, for our patient suggested a benign adenomatoid tumor, which could have initially deescalated surgical intervention. Use of imaging modalities to elucidate between benign and malignant tumors of the scrotum is both challenging and interesting due to unique embryologic origins of paratesticular region allowing for rare malignant pathologies [6]. Further research on imaging guidelines for paratesticular masses is warranted.

Presentations of ES in the male external genitalia are rare, and a review of the literature reveals one reported case of ES within the scrotum but in a 3-year-old-boy with a 2-year history of painless growing mass [5]. The patient had no evidence of metastatic disease at the time of presentation. After surgical resection, due to positive margins, the patient returned for another resection. He underwent chemotherapy with vincristine, doxorubicin, and cyclophosphamide.

On our review of the literature, other primary ES of the male external genitalia has been reported in the penis at least four times. One such case in the penis resulted in a total penile amputation in a young 32-year-old man who subsequently underwent 8 cycles of chemotherapy according to the EuroEwing 99 protocol [4]. The patient was then cancer-free for 3 years before a diagnosis of lung metastases was made, followed by additional chemotherapy and resection. Unfortunately, the patient died due to rapid tumor progression from several recurrences of ES over the next 4 years. Another case of penile ES reported by Toh et al. occurred in a 21-year-old male with a penile lesion, peritoneal mass, and lung metastasis at the time of diagnosis who was later treated with chemotherapy resulting in initial regression but tumor progression 8 months later [13]. Additionally, a 17-year-old with ES of the penis presented with metastasis in the lungs, ribs, lumbar vertebrae, and sacrum at the time of diagnosis [14]. Unfortunately, he died from lung metastasis complications and sepsis 2 months later. Another case was found at the base of the penis but was misdiagnosed as an endocrine disorder before a confirmed ES diagnosis was made at the time of massive lung metastasis [15].

Ewing’s Sarcoma is a cancer most commonly found in children, and much of its management in adults is modeled from pediatric protocols [16]. Due to the rarity of extraossesous ES, there is no consistent algorithm for treatment but it has been proposed that extraskeletal ES and even paratesticular neuroectodermal tumors should be treated with the same ES protocol used in bone tumors [4, 17]. The standard surgical approach to soft tissue sarcomas is a wide excision with negative margins (R0, no microscopic disease); similar principles should be followed. Kushner et al. demonstrated that high doses of cyclophosphamide, doxorubicin, vincristine, ifosfamide, and etoposide generates excellent results in young children and adults [18]. Likewise, a prospective study described the same treatment protocol for both Ewing’s sarcoma and peripheral neuroepithelioma in children and young adults showing no difference in disease-free survival rates for both groups of primitive neuroectodermal tumors [19].

Although not the focus of this discussion, it is worthwhile to note that several cases of primary Ewing’s sarcoma in both children and adults within the bladder, ureter and kidney have been reported and treated with a combination of surgical resection, chemotherapy and radiation [20–23].

In conclusion our case is not only the first known report of paratesticular Ewing Sarcoma in an adult, but it also represents a unique case of an initial apparent adenomatoid tumor on ultrasound that was later found to be primary ES. Although there are several reports of ES in the genitourinary system, there is ultimately one known report of ES in the scrotum and a handful of cases of penile ES. Rare diseases such as this case of a paratesticular Ewing sarcoma in an adult warrant consideration of unique differential diagnoses for scrotal masses. This disease appears to be treated best with surgical resection and adjuvant chemotherapy; however, despite treatment, prognosis is often dismal once metastatic disease develops. Diagnosis and treatment of cases in the adult population is often challenging. Current treatment post-surgical resection in pediatric and adult cases involves adjuvant chemotherapy and possible radiation, according to ES protocols per the National Comprehensive Cancer Network. Future investigation of appropriate diagnostic and management algorithms for extraosseous ES may be considered.

## Data Availability

Data sharing is not applicable to this article as no datasets were generated or analyzed during the current study.

## Abbreviations

ES: Ewing sarcoma
CT: Computer tomography
AFP: Alpha fetoprotein
b-HCG: Beta human chorionic gonadotropin
LDH: Lactate dehydrogenase
FISH: Fluorescence in situ hybridization
MRI: Magnetic resonance imaging

## References

[1] Fitzmaurice C, Dicker D, Pain A, Hamavid H, Moradi-Lakeh M, MacIntyre MF, Allen C, Hansen G, Woodbrook R, Wolfe C, Hamadeh RR, Moore A, Werdecker A, Gessner BD, Te Ao B, McMahon B, Karimkhani C, Yu C, Cooke GS (2015). The global burden of cancer 2013. JAMA Oncol.

[2] Dotan ZA, Tal R, Golijanin D, Snyder ME, Antonescu C, Brennan MF, Russo P (2006). Adult genitourinary sarcoma: the 25-year Memorial Sloan-Kettering experience. J Urol.

[3] Mondaini N, Palli D, Saieva C, Nesi G, Franchi A, Ponchietti R, Tripodi SA, Miracco C, Meliani E, Carini M, Livi L, Zanna I, Trovarelli S, Marinò V, Vignolini G, Pomara G, Orlando VL, Giubilei G, Selli C, Rizzo M (2005). Clinical characteristics and overall survival in genitourinary sarcomas treated with curative intent: a multicenter study. Eur Urol.

[4] Krakorova DA, Halamkova J, Tucek S, Bilek O, Kristek J, Kazda T, Zambo IS, Demlova R, Kiss I (2021). Penis as a primary site of an extraskeletal Ewing sarcoma: a case report. Medicine.

[5] Grimsby GM, Harrison CB (2014). Ewing sarcoma of the scrotum. Urology.

[6] Wald M, Rosevear HM, Mishail A, Sheynkin Y (2009). Unusual scrotal pathology: an overview. Nat Rev Urol.

[7] Amin W, Parwani AV (2009). Adenomatoid tumor of testis. Clin Med Pathol.

[8] Henley JD, Ferry J, Ulbright TM (2000). Miscellaneous rare paratesticular tumors. Semin Diagn Pathol.

[9] AlGhamdi M, AlYami M, Faqeeh S, AlKubeyyer B, AlShabyli N, AlAyed A (2021). Beyond germ cell tumors, unusual testicular and extra-testicular masses and mass-like lesions: MRI and US pictorial review. Clin Imaging.

[10] Shah T, Abu-Sanad O, Marsh H (2016). Role of magnetic resonance imaging in the early diagnosis of paratesticular rhabdomyosarcoma. Ann R Coll Surg Engl.

[11] Sankhe A, Rai P (2022). Imaging in paratesticular lesions. BMJ Case Rep.

[12] Johnson R, Chaljub B, Singh H, Orihuela E (2000). MRI of malignant fibrous histiocytoma of the spermatic cord. J Comput Assist Tomogr.

[13] Toh KL, Tan PH, Cheng WS (1999). Primary extraskeletal Ewing’s sarcoma of the external genitalia. J Urol.

[14] Zheng C, Zhou Y, Luo Y, Zhang H, Tu C, Min L (2020). Case report: primary Ewing sarcoma of the penis with multiple metastases. Front Pediatr.

[15] Song H, Sun N, Zhang W, Huang C (2012). Primary Ewing’s sarcoma/primitive neuroectodermal tumor of the urogenital tract in children. Chin Med J.

[16] Chandran R, Kuruva SP, Chennamaneni R, Bala S, Konatam ML, Gundeti S (2020). Outcomes of adult Ewing sarcoma treated with multimodality therapy: a single-institute experience. South Asian J Cancer.

[17] Galyfos G, Karantzikos GA, Kavouras N, Sianou A, Palogos K, Filis K (2016). Extraosseous Ewing sarcoma: diagnosis, prognosis and optimal management. Indian J Surg.

[18] Kushner BH, Meyers PA, Gerald WL, Healey JH, La Quaglia MP, Boland P, Wollner N, Casper ES, Aledo A, Heller G (1995). Very-high-dose short-term chemotherapy for poor-risk peripheral primitive neuroectodermal tumors, including Ewing’s sarcoma, in children and young adults. J Clin Oncol.

[19] Miser JS, Kinsella TJ, Triche TJ, Tsokos M, Jarosinski P, Forquer R, Wesley R, Magrath I (1987). Ifosfamide with mesna uroprotection and etoposide: an effective regimen in the treatment of recurrent sarcomas and other tumors of children and young adults. J Clin Oncol.

[20] Almeida MF, Patnana M, Korivi BR, Kalhor N, Marcal L (2014). Ewing sarcoma of the kidney: a rare entity. Case Rep Radiol.

[21] Cheng L, Xu Y, Song H, Huang H, Zhuo D (2020). A rare entity of primary Ewing sarcoma in kidney. BMC Surg.

[22] Tonyalı Ş, Yazıcı S, Yeşilırmak A, Ergen A (2016). The Ewing's sarcoma family of tumors of urinary bladder: a case report and review of the literature. Balkan Med J.

[23] Li XX, Bi JB (2019). Ureteral Ewing's sarcoma in an elderly woman: a case report. World J Clin Cases.

